# Rare Presentation of a Common Disease: Graves' Thyrotoxicosis Presented With Non-parathyroid Hypercalcemia and Jaundice

**DOI:** 10.7759/cureus.35206

**Published:** 2023-02-20

**Authors:** Ibrahim Ajwah, Sameerah Alshehri, Fahad Alremthi, Nasser Alahmari

**Affiliations:** 1 Department of Endocrinology, Diabetes and Metabolism, King Abdulaziz Medical City, Ministry of the National Guard-Health Affairs, Riyadh, SAU; 2 Department of Internal Medicine, King Salman Armed Forces Hospital, Ministry of Defense, Tabuk, SAU; 3 Department of Endocrinology, Diabetes and Metabolism, King Abdullah Hospital, Diabetes and Endocrine Center, Ministry of Health (MOH), Bisha, SAU

**Keywords:** parathyroid hormone (pth), auto immune, jaundice, hypercalcemia, graves´disease

## Abstract

Graves' disease (GD) is an autoimmune thyroid disease, which is considered the most common cause of primary hyperthyroidism. GD usually manifests with symptoms such as tremors, palpitations, heat intolerance, weight loss, and specific signs on physical examination (proptosis and pretibial myxedema). However, systemic involvement is also recognized, for example, hepatic involvement in patients with GD may range from asymptomatic laboratory findings of liver function derangement (either transaminases elevations or intrahepatic cholestasis) up to hepatic failure. We describe a rare case of Graves’ thyrotoxicosis presenting with severe cholestasis and non-parathyroid hormone-related hypercalcemia. An extensive evaluation for hepatobiliary causes of cholestasis, including hepatic biopsy, was entirely negative. The patient was successfully treated with methimazole with subsequent clinical and biochemical improvement.

## Introduction

Graves’ disease (GD) is an autoimmune thyroid disease and is considered the most common cause of primary hyperthyroidism [1].

GD usually manifests with symptoms including tremors, palpitations, heat intolerance, weight loss, and specific physical signs (e.g., proptosis and pretibial myxedema) [2].

However, systematic involvement is also known to occur; for example, hepatic involvement in patients with GD may range from asymptomatic laboratory findings of liver function derangement (either transaminase elevation or intrahepatic cholestasis) to hepatic failure [3].

Hyperthyroidism is known to be associated with non-parathyroid hypercalcemia, and asymptomatic hypercalcemia is reported in around one-fifth of cases of hyperthyroidism [4].

We report a case of GD presented with atrial fibrillation, electrolyte imbalance, and cholestatic jaundice.

This article was previously presented as a poster at the AACE Communities MENA - Middle East & North Africa Conference on November 11-13, 2022.

## Case presentation

A 22-year-old single male with a past medical history of bronchial asthma presented with shortness of breath, palpitations, and jaundice with severe itching and dark urine. There was no abdominal pain, loose stool, nausea, or vomiting.

Apart from bronchial asthma, his past history was unremarkable. He denied any contact with sick people with an upper respiratory infection, COVID-19, or pneumonia, previous history of hepatitis, or high-risk behaviors such as illicit drug abuse or unprotected sexual activity. In addition, he denied any exposure to hepatotoxic drugs or alcohol consumption.

There were no other systemic complaints such as joint pain, muscle weakness, or skin rash. 

He was in his usual state of health until he noticed that his sclera started to become yellow, with progressive darkening of his urine as well as generalized itching for two weeks, followed by rapid weight loss (16 kg) over two weeks. Suddenly, he developed palpitations associated with shortness of breath and sought a visit to the emergency department.

On admission, he appeared ill but was conscious and oriented, deeply jaundiced, and vitally stable except for tachycardia (blood pressure 142/84 mmHg, heart rate 135 beats/min, respiratory rate 23/min, oxygen saturation 99% in room air, BMI 23.2).

Examination of the chest and cardiovascular system was unremarkable. Abdominal examination revealed a soft abdomen with mild tenderness over the right hypochondrium without organomegaly or palpable lymphadenopathy.

No goiter or signs of Graves’ orbitopathy were observed, and there were mild bilateral tremors.

His laboratory investigation results were as follows:

Normal blood count and differential, an electrolyte disturbance in the form of non-parathyroid-related moderate hypercalcemia (adjusted calcium: 3.4 mmol/L, parathyroid hormone [PTH]: 0.6 pmol/L [normal PTH 2.65-12.73 pmol/L]) and hypokalemia (serum potassium: 2.8 mmol/L) associated with ECG changes, initially showing an atrial fibrillation rhythm with subsequent restoration of a normal sinus rhythm with sinus tachycardia. Liver function tests demonstrated a cholestatic pattern (total bilirubin: 674.9 µmol/L, direct bilirubin: 533.6 µmol/L).

Based on his presenting symptoms, thyroid function tests were ordered and showed undetectable thyroid-stimulating hormone (TSH) (<0.01 mIU/L) and elevated tetraiodothyronine (thyroxin, T4) and triiodothyronine (T3) (T4: 36.25 pmol/L [9.00-19.00 pmol/L], T3: 16.51 pmol/L [2.90-4.90 pmol/L]), suggestive of hyperthyroidism. As part of the hyperthyroidism examination, a 99mTc thyroid uptake scan was ordered and showed a high uptake at 19.2% (normal 1-4%). Furthermore, thyroid autoantibody screening revealed a positive result for thyroid receptor antibodies (4.8+ IU/L) (Table 1).

**Table 1:** Thyroid autoantibody

Exam name	Result	Reference value
Thyroglobulin antibodies	214.70 IU/mL	5-100 IU/mL is normal, >100 IU/mL is positive
Thyroid peroxidase antibodies (TPO antibodies)	312.90 IU/mL	1-16 IU/mL is normal, >16 IU/mL is positive
Thyroid-stimulating hormone (TSH) receptor antibodies	4.8 IU/L	<1.8 IU/L

Workup for his cholestatic jaundice included screening for viral serology (hepatitis A virus [HAV], hepatitis B virus [HBV], hepatitis C virus [HCV], cytomegalovirus [CMV], herpes simplex virus [HSV], varicella-zoster [VZ], Epstein Barr virus [EBV]), autoimmune markers (antinuclear antibody [ANA], immunoglobulin G [IgG], anti-liver kidney microsome type 1 (anti-LKM-1) antibody, antimitochondrial antibody [AMA], anti-smooth muscle antibody [ASMA]), hemochromatosis (transpiration saturation), Wilson’s disease (ceruloplasmin), and celiac disease (tissue transglutaminase [TTG]), as well as toxicology screening; all of them showed negative results.

Abdominal ultrasound showed features suggestive of parenchymal liver disease with no biliary dilatation and normal common bile duct (CBD) and gallbladder. The major portal and hepatic veins were patent with normal Doppler waveform and flow direction. The spleen was normal in size.

Magnetic resonance cholangiopancreatography (MRCP) showed normal CBD and intra- and extrahepatic biliary duct. The gallbladder was unremarkable, and no focal hepatic lesion was identified.

A liver biopsy revealed peripheral cholestasis not associated with obstruction or inflammation.

Therefore, medical management included the correction of hypokalemia with intravenous potassium replacement, management of moderate hypercalcemia with normal saline and calcitonin, and management of GD with methimazole initiated at a dose of 15 mg once daily with atenolol at 25 mg orally.

One month later, he became asymptomatic and gained weight, and his jaundice started to resolve. This symptomatic improvement was associated with the normalization of the calcium and potassium levels and reduction in the bilirubin concentration, and at this point, methimazole was tapered down to 5 mg (Tables 2, 3).

**Table 2:** Liver function in relation to hyperthyroidism status

Exam name	Before treatment	After treatment	Reference value
Thyroid-stimulating hormone (TSH)	<0.01	<0.01	0.35-4.94 mIU/L
Free T4	36.25	10.2	9.00-19.00 pmol/L
Free T3	16.51	2.6	2.90-4.90 pmol/L
Bilirubin (total)	674.9	61.6	~20.5 µmol/L
Bilirubin (direct)	533.6	46.7	~8.6 µmol/L
Alkaline phosphatase	502	190	40-150 U/L
Alanine transaminase (ALT)	65	44	5-55 U/L
Aspartate aminotransferase (AST)	69	44	5-34 U/L

**Table 3:** Calcium and potassium levels in relation to hyperthyroidism status

Exam name	Before treatment	After treatment	Reference value
Thyroid-stimulating hormone (TSH)	<0.01	<0.01	0.35-4.94 mIU/L
Free T4	36.25	10.2	9.00-19.00 pmol/L
Free T3	16.51	2.6	2.90-4.90 pmol/L
Phosphorus	0.92	1.3	0.74-1.52 mmol/L
Adjusted calcium	3.4	2.37	2.1-2.55 mmol/L
Potassium	2.8	4.1	3.5-5.1 mmol/L

## Discussion

We report the case of a young male who presented with atrial fibrillation, hypercalcemia, hypokalemia, and cholestatic jaundice.

Liver function abnormality is a common finding in hyperthyroid patients (60%), and abnormal alanine aminotransferase is reported in 44% of cases. Furthermore, a slight elevation of serum bilirubin may be observed in GD patients. However, severe intrahepatic cholestasis is considered an unusual presentation for GD [5].

Nevertheless, jaundice could be a sign of hyperthyroidism of any etiology including GD. In addition, hyperthyroidism can worsen preexisting liver disease. For example, Thompson et al. reported a case of reversible worsening of liver function in a patient known to have primary biliary cirrhosis (PBC), who had complete resolution of jaundice after controlling his hyperthyroidism [6].

In our case, the patient was not known to have preexisting liver disease, and at the initial stage of the management, we aimed to exclude primary hepatobiliary disease before associating his hyperbilirubinemia state with hyperthyroidism, similar to biliary obstruction, acute hepatitis (viral and autoimmune), Wilson’s disease, hemochromatosis, and drug-induced hepatitis.

Based on the differential diagnosis, transabdominal ultrasonography and MRCP were performed to exclude biliary obstruction due to stone or biliary sclerosis, and both showed normal results. PBC has been associated with autoimmune conditions. Although the association between PBC and autoimmune hypothyroidism is well documented, the occurrence of PBC and GD is rarely reported. Nevertheless, in clinical practice, both coexisting and pre-existing thyroid abnormalities have been reported. Yaşar et al. described a case of a 51-year-old female who presented with hyperthyroid symptoms and cholestatic pattern liver function abnormalities due to GD accompanied with PBC diagnosed at the same time. Furthermore, in a case of late-onset PBC, Suzuki et al. reported the case of a 62-year-old man known to have GD who developed PBC years after GD diagnosis [7,8].

The most common cause of elevated calcium levels is primary hyperparathyroidism; on the other hand, only 15-20% of cases of hyperthyroidism involved hypercalcemia [4].

In our case, hypercalcemia was caused by thyrotoxicosis without the presence of other contributing factors such as PTH elevation or vitamin D intoxication. Additionally, the complete normalization of serum-adjusted calcium levels along with the control of hyperthyroidism and normalization of thyroid function tests were observed.

## Conclusions

Palpitations, heat intolerance, weight loss, and menstrual abnormalities are the common symptoms of hyperthyroidism of any cause including GD. The development of jaundice in patients with GD may be attributed to hyperthyroidism itself, autoimmune hepatitis, or viral hepatitis. Anti-thyroid drugs can be administered with careful monitoring to treat hyperthyroidism in the case of cholestasis.
